# Cold Exposure Affects Lipid Metabolism, Fatty Acids Composition and Transcription in Pig Skeletal Muscle

**DOI:** 10.3389/fphys.2021.748801

**Published:** 2021-10-06

**Authors:** Ziye Xu, Wentao Chen, Liyi Wang, Yanbing Zhou, Qiuyun Nong, Teresa G. Valencak, Yizhen Wang, Jintang Xie, Tizhong Shan

**Affiliations:** ^1^College of Animal Sciences, Zhejiang University, Hangzhou, China; ^2^Key Laboratory of Molecular Animal Nutrition, Ministry of Education, Zhejiang University, Hangzhou, China; ^3^Key Laboratory of Animal Feed and Nutrition of Zhejiang Province, Hangzhou, China; ^4^Shandong Chunteng Food Co., Ltd., Zaozhuang, China

**Keywords:** cold exposure, meat quality, fatty acid, transcriptome, pig, skeletal muscle

## Abstract

Cold exposure promotes glucose oxidation and modulates the lipid metabolism in adipose tissue, but it is still not fully clear whether cold exposure could affect meat quality and fatty acid metabolism in skeletal muscle of pig *in vivo*. Here, we kept finishing pigs under cold or room temperature overnight and determined the effects of cold exposure on meat quality, fatty acids composition and transcriptional changes in skeletal muscle of pigs. We found that cold exposure significantly reduced the meat colour_24 h_ and pH_24 h_, without affecting carcass characteristics and other meat quality traits. Considerable changes were found in the proportions of individual fatty acids and the total content of saturated fatty acid, polyunsaturated fatty acids, monounsaturated fatty acid and n3-fatty acids. RNA-seq results showed upregulated fatty acid biosynthesis genes and downregulated mitochondrial beta-oxidation genes. The lipid metabolism in cold-treated longissimus dorsi muscle might be regulated by functions of the lipoprotein particle, the extracellular matrix, and the PPAR signaling pathways. Our study revealed the potential of cold exposure to regulate the lipid metabolism and fatty acid composition in skeletal muscle of farmed animals.

## Introduction

Emerging evidences have indicated that cold exposure plays a crucial role in whole-body lipid metabolism, including reduced plasma triglyceride (TG) concentrations by activating brown adipose tissue (BAT)-mediated non-shivering thermogenesis (Bartelt et al., 2011), reversed cholesterol transport by high-density lipoprotein (HDL) particles (Bartelt et al., 2017) and increased conversion of cholesterol to bile acids (BAs) (Worthmann et al., 2017). Cold exposure also has multiple effects on hepatic lipid metabolism and microbiome composition, which collaborates with thermogenic BAT in maintaining whole-body metabolic homeostasis in mice (Ziêtak et al., 2016; Grefhorst et al., 2018). Our previous results revealed that short-term cold exposure induces significant changes in lipid dynamics and gene expression pathways in inguinal WAT (iWAT) (Xu Z. Y. et al., 2019). These results demonstrate that cold stimulation is an effective way to modulate the systemic metabolic homeostasis, especially glucose and lipid metabolism.

Pork is the most widely consumed meat in the world accounting for over 36% of the world-wide meat intake (Dugan et al., 2015). Studies on improving pork quality and nutritive values have received wide attention over the recent 10 years, mainly in response to the stricter quality controls and increased customer awareness (Grunert et al., 2004). Pork quality generally is evaluated through water-holding capacity, meat color, pH, fat content and oxidative stability, and its nutritive value is mainly determined by fatty acid profile, especially the n-3 PUFA proportion (Rosenvold and Andersen, 2003; Hocquette et al., 2010). Intramuscular fat (IMF), also known as intramuscular triglycerides, which refers to adipose tissue located in myofibers (Liu et al., 2019). In pork production, IMF has been recognized as an important meat-quality trait of pigs owning to influencing the shearing force, tenderness and juicy flavor (Zhang et al., 2021). Moreover, IMF accumulation in muscle is related to diseases such as insulin resistance and type 2 diabetes (Buras et al., 2019). Scientists have put forward various strategies to improve pork quality and nutritive values in the past few decades, including improving the genetic background of pig breeds, optimizing nutrient supply and the production systems (Rosenvold and Andersen, 2003; Hocquette et al., 2010). However, the pork industry is challenged because many of these efforts were imperceptible to the consumer with at the same time causing increased production costs.

Improving pre-slaughtering conditions, such as housing and exercise, could be a novel and effective method to amend the sensory and nutrient quality of pork (Ngapo and Gariepy, 2008). As temperature changes are easily perceived by mammals, we hypothesized that meat quality might be improved by acute cold exposure before slaughter. Unlike rodents, pigs reportedly have no BAT and defending their body temperature depends on skeletal muscle shivering as a primary source of heat production when exposed to cold (Hou et al., 2017; Blondin and Haman, 2018). The skeletal musculature might produce heat through both shivering and non-shivering thermogenesis, both of which influence mitochondrial energetics and remodels fat content and composition (Blondin and Haman, 2018). In addition, cold exposure increases whole-body energy expenditure and improves glucose metabolism by inducing sympathetic nervous system activity and recruiting brown adipocytes as shown recently from human (Van Der Lans et al., 2013; Iwen et al., 2017). By reconstitution of the functional uncoupling protein 1 (UCP1) gene in white adipose tissue of pigs, a browning-like adipocyte of BAT was induced resulting in decreased fat deposition, increased lean percentage and altered lipid metabolism in adipose (Pan et al., 2019). In pigs, UCP3 was shown to mediate some non-shivering thermogenic activity (Lin et al., 2017). Based on these previous results, we supposed that cold exposure might influence meat quality in the skeletal muscle of pigs both directly and indirectly.

In the present study, we kept growing-finishing pigs under cold (5–7°C) or room temperature (22–25°C) overnight (14 h) and investigated the effects of acute cold exposure on carcass indicators, enzyme activity, fatty acid composition, and gene expression profiles in longissimus dorsi muscle (LDM). We revealed cold exposure plays a key role in pork lipid metabolism and fatty acid profiles. Moreover, these results were accompanied by changes in transcriptional dynamics *in vivo*, especially fatty acid oxidation and fat biosynthesis. Our results point out the importance of pre-slaughter temperature conditions for fatty acid metabolism of pork.

## Materials and Methods

### Animals and Experimental Design

All procedures were approved by the University of Zhejiang Institutional Animal Care and Use Committee. The ethical committee number for the study is ZJU20170466. Duroc × Landrace × Yorkshire (DLY) boars were raised in Shandong Chunteng Food Co., Ltd., (Tengzhou city, Shangdong, China) and fed twice a day with the same diet with 67% of corn, 21% of soybean meal, 8% of wheat bran and 4% series of pig premixed feed and provided free access to water under similar environmental. At slaughter weight (120–125 kg,6.0–6.5 months), twelve finishing pigs were randomly selected and used to investigate the influence of acute cold exposure on pork meat quality and fatty acids composition.

These pigs were divided into two groups randomly and each contains six animals. These two groups experimental animals were placed at room temperature (RT, 22–25°C) and under cold conditions (COLD, 5–7°C) overnight (14 h), respectively. During the 14 h, all pigs were fasted but free access to water. These pigs were weighed and sampled immediately after short-term cold exposure.

### Slaughtering, Carcass and Meat Quality Measurements

Pigs were slaughtered in a commercial abattoir by exsanguination after electric stunning (90∼100 V, 0.9–1.0 A, 50 Hz). After that, the pigs were immediately hoisted for bleeding and dehairing. Evisceration was completed about 20 min post mortem.

The carcass was split longitudinally after the head, legs, tail, and viscera were removed. Carcass traits were measured by using standard methodology for testing carcass traits in lean-type pig (Gu et al., 2019). Both the left and right hot carcass weights were recorded. Carcass yield was calculated by hot carcass weight/preslaughter weight ×100% (Gu et al., 2019). Body length was measured as the distance from the anterior edge of the first cervical vertebra to the anterior edge of the pubis (Gu et al., 2019). Skin thickness was assessed at the 6rd/7th rib of the centerline of the carcass by using a Vernier caliper (Li et al., 2020). Backfat thickness was determined by calculate the average scores of three regions of the right carcass sides (first- and last-rib, and last-lumbar) (Li et al., 2018). Simultaneously, samples of LDM from the right side of each carcass were collected and rapidly frozen in liquid nitrogen and subsequently stored at −80°C for fatty acid composition and RNA-seq analysis.

Meat quality measurements including marbling, pH_45 min_, pH_24 h_, drip loss (24 h), meat color_45 min_ and meat color_24 h_ were carried out in LDM obtained from the 3rd to 11th rib. Subjective marbling was scored from a mean of scores made by three people using the National Pork Producer Council (NPPC) standards as previous described (Xu X. et al., 2019). The pH_45 min_ values were determined on LDM at 45 min postmortem using a portable pH meter (pH-STAR, MATTHAUS, German), previously calibrated with pH 4.6 and 7.0 buffers (Xu X. et al., 2019). The meat color_45 min_ values were measured from a mean of three random readings on LDM at 45 min postmortem using a portable chromameter (opto-STAR, MATTHAUS, German), which was calibrated with a white tile according to the manufacturer’s manual (Xu X. et al., 2019). Samples were stored at 4°C for 24 h, and then pH_24 h_ and meat color_24 h_ were measured in the same way. Drip loss was measured using the hanging bag method (Honikel, 1998), 2.5 cm thick loin chops were taken from LDM at the third and fourth lumbar vertebrae after slaughter. The initial weights of these loin chops were collected, and meat samples were reweighed after stored for 24 h at 4°C to collect the terminal weights. Drip loss was calculated as the percentage of weight lost over the 24 h period. LDM samples were homogenized into freeze-dried powder and then weighed. Intramuscular fat (IMF) content was measured by determining the crude fat of LDM by using Soxhlet Extraction with petroleum ether (Tyra and Żak, 2012). Inosinic acid content was measured described by Xu X. et al. (2019). The IMF and inosinic acid contents were indicated by the weights of fat or inosinic acid, respectively, in per 100 g freeze-dried LDM (g/100 g).

### Enzyme Activities

Longissimus dorsi muscle samples were lysed in phosphate buffered saline (PBS) and the supernatant was obtained by centrifugation at 2,000 rpm for 10 min and used for subsequent enzyme activities measurements. The BCA Protein Assay Kit (Thermo Fisher Scientific) was used to measure protein concentrations. The contents of triglyceride (TG) and non-esterified free fatty acids (NEFA), and the enzyme activities of lactate dehydrogenase (LDH), succinate dehydrogenase (SDH), malate dehydrogenase (MDH), malondialdehyde (MDA), lipid peroxide (LPO), total antioxidant capacity (T-AOC), glutathione peroxidase (GSH-Px), catalase (CAT) and peroxidase (POD) in COLD or RT group were measured using commercially available kits according to the manufacturer’s instructions (Nanjing Jiancheng Bioengineering Institute, Nanjing, China).

### Analysis of the Fatty Acid Composition

Lipids in the LDM were extracted and hydrolyzed in 2 mL KOH-methanol to obtain the free fatty acid mixture. The free fatty acid mixture was esterified in 2 mL BF3-methanol solution to obtain fatty acid methyl esters. Next, 800 μL fatty acid methyl esters were separated and analyzed with a GC-2010 plus gas chromatograph (Shimadzu, Japan). By comparing the retention times of the peaks with the known standards (Sigma, United States), fatty acids contents could be identified.

### Analysis of the Amino Acid Composition

About 150 mg of the dried LDM sample were weighed and put into a glass cylinder before 15 mL of 6 molar HCl was added. After adding nitrogen and sealing, the mixture was hydrolyzed at 110°C for 22–24 h. Subsequently, the hydrolysate was transferred to a 50 mL volumetric flask and diluted into a calibration tail with ultrapure water. The solution was filtered using a 0.45 μm membrane filter into an autosampler vial before amino acid analysis with an L-8900 amino acid analyzer (HI-TACHI, Japan).

### RNA Isolation, Library Construction, RNA-Seq Analysis and Quantitative Real-Time

#### PCR

RNA extraction, library construction, RNA-seq analysis and quantitative real-time PCR (qPCR) of LDM samples from RT and cold-treated pigs were performed as previously published methods (Xu Z. et al., 2019; Xu et al., 2020). Briefly, total RNA was extracted using the Total RNA Extractor (TRIzol) Kit (B511311, Sangon, China) and the quality of the RNA samples was examined with a NanoDrop 2000 spectrophotometer (Agilent Technologies, Santa Clara, CA, United States). A total amount of 2-μg RNA per sample was used for library preparation. Subsequently, paired-end sequencing of the library was performed on HiSeq XTen sequencers (Illumina, San Diego, CA, United States). FastQC (version 0.11.2) was used to evaluate the quality of the sequenced data. Trimmomatic (version 0.36) and HISAT2 (version 2.0) were applied to filter raw reads and mapped to the reference genome, respectively. Package DESeq2 (version 1.12.4) was used to identify differentially expressed genes (DEGs) between the two groups. Genes with *p* value < 0.05 and | Log2 (fold change) | > 1 were considered significant DEGs. QPCR was performed with an Applied Biosystems StepOnePlus^TM^ Real-Time PCR System using SYBR Green Master Mix (Roche, Indianapolis, IN, United States). The relative changes in gene expression were analyzed by using the 2^–ΔΔCT^ method.

#### Pathway-Enrichment Assay

Gene Ontology (GO) functional analysis and KEGG pathway analysis were performed as previously published methods (Xu Z. et al., 2019; Xu et al., 2020). Briefly, DEGs are subjected to GO biological process (BP) and KEGG pathway enrichment analysis using the packages clusterProfiler and org.Ss.eg.db. *P* value < 0.05 was defined as statistical significance. Enriched terms and pathways were visualized by the barplot and cnetplot function.

### Statistical Analysis

Data on carcass and meat characteristics, enzyme activities and fatty acid composition were presented as the mean ± SEM. Comparisons were made by unpaired two-tailed Student’s *t*-tests. Differences between groups were considered statistically significant at *p* < 0.05.

## Results

### Cold Exposure Induced Alterations in Meat Characteristics and Enzyme Activities

The carcass and meat characteristics from immediate post-mortem are given in Table 1. There were no significant differences in body weights (BW), carcass weights, body lengths, skin thickness, and mean backfat thickness between cold-treated and RT pigs (Table 1). Next, we found that overnight cold exposure significantly reduced the meat colour_24 h_ (*p* = 0.046) and pH_24 h_ (*p* = 0.009), without affecting the meat colour_45 min_, pH_45 min_, marbling and drip loss of pigs (Table 1). Moreover, the content of IMF had an increasing tendency (*p* = 0.061) in cold-treated meat (Table 1). While the flavor substance and the content of inosinic acid, were not affected by cold exposure (*p* > 0.05, Table 1). Besides, we neither found a pH_24 h_ value above 6.0 nor a pH_45 min_ value below 5.8 in this study, indicating no dark, firm and dry (DFD) or pale, soft and exudative (PSE) meat (Table 1).

**TABLE 1:** Effects of cold exposure on carcass characteristics, meat quality traits and flavor substances of LDM in pigs.

**Variable**	**RT**		**COLD**		
	Mean	SEM	Mean	SEM	*P*-value
BW (kg) ^1^	121.917	2.491	124.250	5.261	0.697
Carcass weight (kg)	94.817	2.485	97.183	4.106	0.633
Carcass yield	0.777	0.007	0.784	0.021	0.777
Body length (cm)	108.333	1.498	112.167	3.027	0.283
Skin thickness (mm)	3.342	0.107	3.135	0.318	0.552
Backfat thickness (mm)	33.247	2.604	33.647	2.160	0.908
Marbling	1.000	0.000	1.167	0.167	0.341
Meat colour45 min ^2^	86.361	0.983	86.589	1.333	0.893
Meat colour24 h ^3^	72.778	1.593	68.400	1.083	0.046*
pH45 min ^4^	6.389	0.085	6.234	0.091	0.241
pH24 h ^5^	5.667	0.056	5.453	0.034	0.009**
Drip loss (%)	2.047	0.065	2.071	0.091	0.837
IMF (g/100 g) ^6^	12.150	0.274	13.117	0.368	0.061
Inosinic acid (g/100 g) ^7^	0.830	0.034	0.838	0.034	0.866

Statistical effect of cold exposure on carcass and meat characteristics of pigs were analyzed by two-tailed Student’s *t*-test (n=6). SEM, standard error of means. *P < 0.05, **P < 0.01. RT, pigs at room temperature of 22–25°C; COLD, pigs at cold temperature of 5–7°C.

*^1^BW, body weight.*

*^2^Meat color45 min, meat color measured 45 min after slaughter.*

*^3^Meat color24 h, meat color measured 24 h after slaughter.*

*^4^pH45 min, pH value measured 45 min after slaughter.*

*^5^pH24 h, pH value measured 24 h after slaughter.*

*^6^IMF, intramuscular fat. The IMF content was indicated by the weight of fat (g) in pre 100 g freeze-dried LDM (g/100 g).*

*^7^The inosinic acid content was indicated by the weights of inosinic acid (g) in pre 100 g freeze-dried LDM (g/100 g).*

**FIGURE 1:**
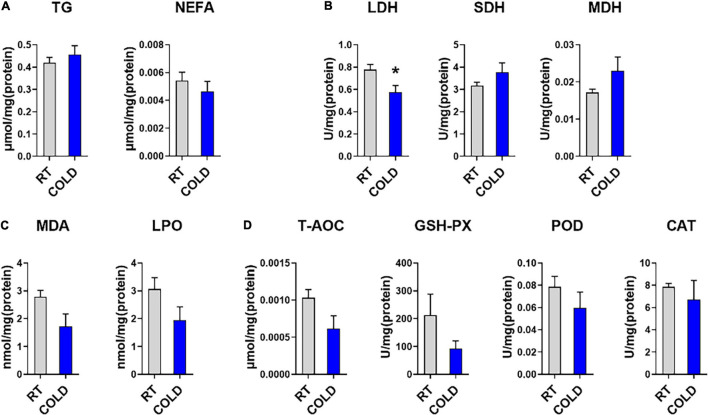
Effects of cold exposure on lipid metabolism and various enzymes activities. **(A)** Triglyceride (TG) and non-esterified free fatty acids (NEFA) levels in LDM of pigs from room temperature (RT) or cold condition (COLD). **(B)** The activities of oxidoreductases, including Lactate dehydrogenase (LDH), succinate dehydrogenase (SDH), malate dehydrogenase (MDH). **(C)** The levels of lipid peroxide contents, including malondialdehyde (MDA), lipid peroxidation (LPO). **(D)** The levels of antioxidant non-enzymatic activity, total antioxidant capacity (T-AOC), and enzymatic activities, including glutathione peroxidase (GSH-Px), peroxidase (POD), catalase (CAT). *n* = 5. Error bars represent S.E.M. *P < 0.05, two-tailed Student’s *t*-test.

To further determine the effect of cold exposure on IMF content, we also measured the level of TG and NEFA in pork. Consistent with change trend of IMF content, the content of TG showed an increased trend in cold-treated LDM, while, the content of NEFA showed a decreased trend (Figure 1A). To explore the changes in oxidative stability and antioxidant enzymes stability in skeletal muscle from COLD pigs, we measured enzyme activities associated with oxidation-reduction, lipid oxidative, and antioxidation (Figures 1B–D). The activity of LDH, which is responsible for lactic acid production in glycolysis, was significantly inhibited by cold exposure. These results suggest cold exposure may inhibit glycolysis capacities in LDM. The activities of oxidative enzymes, such as SDH and MDH, showed no change (Figure 1B). Besides, the qPCR results indicated the mRNA level of *MYH2* was significantly downregulated, while the mRNA level of *MYH4* was significantly upregulated (Supplementary Figure 1A). Slightly decreased lipid peroxidation related enzymes (MDA, and LPO) were found in COLD pigs (Figure 1C), suggesting that cold exposure prior to slaughter might improve the quality and shelf-life of meats by lipid oxidative stability. COLD pigs also had differentially expressed antioxidant-related enzymes (T-AOC, GSH-PX, CAT, and POD) (Figure 1D). Taken together, cold exposure may have a positive impact on meat quality and flavor through increasing IMF content, improving oxidative capacity, inhibiting lipid peroxidation, although it did not reach statistical significance.

### Cold Exposure Changed the Composition and Content of Fatty Acids

**FIGURE 2:**
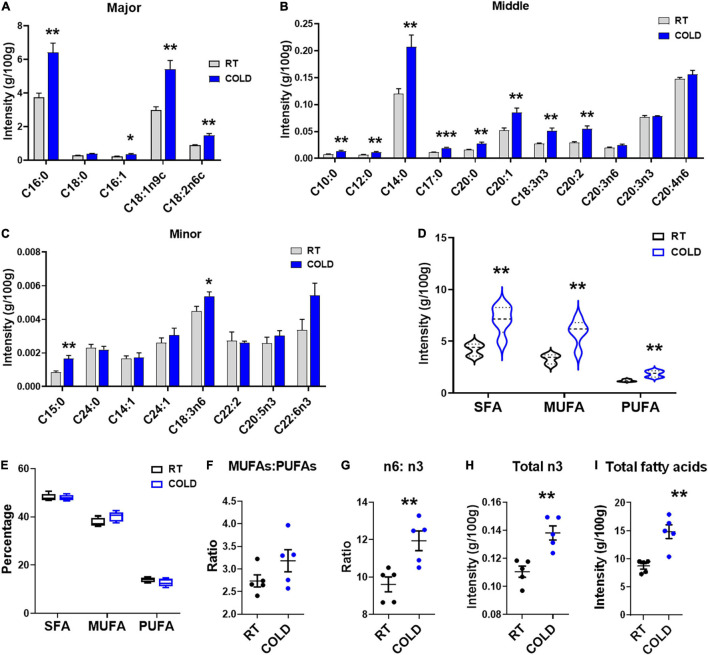
Cold exposure changed the composition and proportions of fatty acids. Fatty acid composition analyses of LDM isolated from pigs maintained at room temperature (RT, 22–25°C) or in cold (5–7°C) for 14 h. **(A–C)** The concentration of individual fatty acids in LDM from COLD and RT pigs. Fatty acids are divided into major **(A)**, middle **(B)** and minor **(C)** species based on abundance. Fatty acids are sorted by a degree of saturation. **(D,E)** The contents **(D)** and percentages **(E)** of total SFAs, MUFAs and PUFAs in LDM from COLD and RT pigs. SFAs, saturated fatty acids; MUFAs, monounsaturated fatty acids; PUFAs, polyunsaturated fatty acids containing two or three to six double bonds. **(F,G)** The ratio of MUFAs: PUFAs **(F)** and n6-fatty acids: n3-fatty acids (n6: n3) **(G)** in LDM from COLD and RT pigs. **(H,I)** The contents of total n3-fatty acids **(H)** and total individual fatty acids **(I)**. *n* = 5. Error bars represent S.E.M. *P < 0.05, **P < 0.01, ***P < 0.001, two-tailed Student’s *t*-test.

We further explored overall fatty acid composition in LDM of COLD pigs compared to RT pigs (Figure 2). Absolute proportions showed that cold exposure induced extensive increases in the following fatty acids: palmitic acid (C16:0), palmitoleic acid (C16:1), oleic acid (C18:1n9c), linoleic acid (C18:2n-6c), capric acid (C10:0), lauric acid (C12:0), myristic acid (C14:0), margaric acid (C17:0), arachidic acid (C20:0), eicosenic acid (C20:1), α-linolenic (C18:3n-3), eicosadienoic acid (C20:2), pentadecanoic acid (C15:0) and γ-linolenic (C18:3n6) (Figures 2A–C). Notably, the saturated fatty acids (SFAs), including margaric acid (C17:0), palmitic acid (C16:0), capric acid (C10:0), lauric acid (C12:0), myristic acid (C14:0), eicosadienoic acid (C20:2) and pentadecanoic acid (C15:0) were largely increased in COLD pigs (Figures 2A–C). In line with the increases in individual fatty acid, the total contents of saturated fatty acids (SFAs)and unsaturated fatty acids (UFAs) were significantly elevated in COLD LDM (Figure 2D). We further analyzed the percentages of total SFAs, monounsaturated fatty acids (MUFAs) and polyunsaturated fatty acids (PUFAs), respectively, and found no difference (Figure 2E). Besides, we calculated the ratio of MUFAs: PUFAs, the ratio of n6-fatty acids: n3-fatty acids (n6: n3) and the total content of n3-fatty acids (Figures 2F–I), all known for improving human health. Cold exposure treatment did not affect the ratio of MUFAs: PUFAs (Figure 2F), but significantly increased the ratio of n6: n3 in LDM (Figure 2G). However, the total content of n3-fatty acids was significantly increased by cold exposure (Figure 2H). The total content of individual fatty acids was also dramatically elevated by cold exposure treatment (Figure 2I). These results suggest that cold exposure induced considerable alterations in the composition and content of fatty acids in LDM.

### Cold Exposure Changed the Transcriptome Profiles of Longissimus Dorsi Muscle in Pigs

**FIGURE 3:**
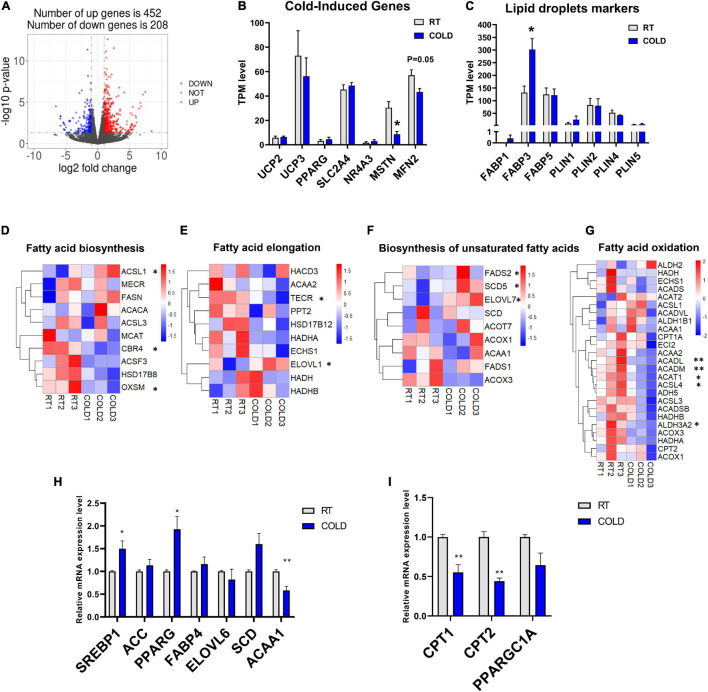
Cold exposure changed the transcriptome profile of LDM in pigs. **(A)** Volcano plot of differently expressed genes (DEGs) expression patterns were illustrated. Red denotes upregulated genes in cold pigs; blue denotes downregulated genes in cold pigs; gray denotes genes with no significant changes. **(B,C)** Transcripts per million (TPM) expression values of cold-induced genes and lipid droplets markers LDM from cold-treated and RT pigs were shown to estimate relative gene expression abundance (*n* = 3). Error bars represent SEM. *P < 0.05, **P < 0.01, two-tailed Student’s *t*-test. **(D–G)** Heatmaps of the TPM expression values of selected fatty acid biosynthesis, fatty acid elongation, biosynthesis of unsaturated fatty acids, fatty acid degradation regulated genes from the RNA-seq dataset. *P < 0.05, **P < 0.01. **(H–I)** qPCR validation of the expression of genes related to fatty acid metabolism in LDM from cold-treated and RT pigs.

To explore how the LDM transcriptome is altered upon cold exposure, we next utilized RNA-seq to map the transcriptional changes. A total of 660 DEGs were identified in the RT and COLD group using the filter criteria of | Log2 (fold change) | > 1 and *p*-value < 0.05. Of these DEGs, 452 were up-regulated and 208 were down-regulated by cold exposure (Figure 3A). The mRNA expression levels of several genes reported previously as having altered expression upon cold exposure in skeletal muscle, including *UCP2, UCP3, PPARG, SLC2A4, NR4A3, MSTN, MFN2*, were reflected by TPM (Transcripts Per Kilobase of exon model per Million mapped read) values (Figures 3B,C). Interestingly, only myogenesis related genes (*MSTN, MFN2*) were decreased by cold exposure in LDM of pigs (Figure 3B). These genes (*UCP2, UCP3, FGF21, SLC2A4, NR4A3*) were not significantly altered in COLD groups (Figure 3B). We also analyzed the expression levels of lipid droplets markers (*FABP1, FABP3, FABP5, PLIN1, PLIN2, PLIN4, PLIN5*) and found that cold exposure significantly elevated the expression level of FABP3 (Figure 3C). Expression levels of genes involved in the main pathways associated to fatty acid metabolism are given in Figure 3. And include fatty acid biosynthesis (Figure 3D), fatty acid elongation (Figure 3E), biosynthesis of unsaturated fatty acids (Figure 3F) and fatty acid degradation (Figure 3G). These significantly altered genes (*CBR4, ACSL1, OXSM, TECR, ELOVL1, SCD5*, *FADS2, ELOVL7, ACADL, ACADM, ACAT1, ACASL4, ALDH3A2*) are highlighted in the heatmaps (Figures 3D–G). The expression levels of *CBR4*, *ACSL1* and *OXSM*, which participate in the biosynthesis of fatty acids in mitochondria (Zhang et al., 2005; Venkatesan et al., 2014), *FADS2*, which regulates the unsaturation of fatty acids, *ELOVL1*, *TECR* and *ELOVL7* which catalyze the long-chain fatty acids elongation (Moon and Horton, 2003; Naganuma et al., 2011), *SCD5*, which catalyzes the formation of monounsaturated fatty acids, was significantly altered by cold exposure. And the expression levels of mitochondrial beta-oxidation related genes (*ACADL, ACADM, ACAT1*) were significantly suppressed in COLD LDM. The RNA-seq results were further confirmed by qPCR on several key genes including *ACC*, *FABP4*, *SREBP1*, *SCD*, *ACAA1*, *PPARG*, *ELOVL6 CPT1*, *CPT2*, and *PPARGC1A* (Figures 3H,I). Taken together, we found extensive changes in the transcriptome of LDM in pigs in response to overnight cold compared with RT. Notably, the fatty acid anabolism related genes were activated, while fatty acid catabolism related genes were inhibited in COLD LDM.

### Cold Exposure Induced Alterations in Apolipoprotein Function

**FIGURE 4:**
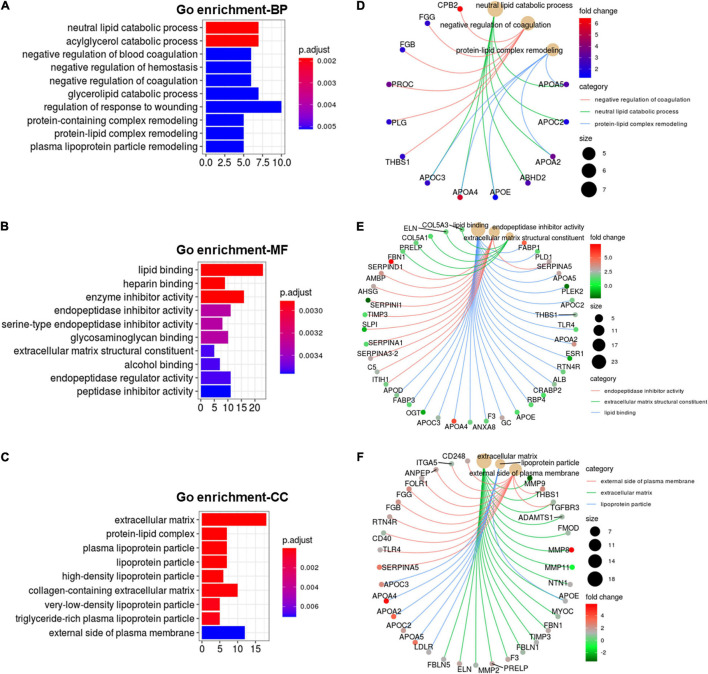
Cold exposure induced alterations in apolipoprotein function. **(A-C)** GO annotation for DEGs in the category of biological process (BP) **(A)**, molecular function (MF) **(B)**, and cell component (CC) **(C)**. **(D–F)** The cnetplot depicts the linkages of the selected GO terms in the category of BP **(D)**, MF **(E)**, CC **(F)** and genes involved in these terms as a network.

Functional enrichment analysis of cold-induced DEGs were carried out using GO enrichment analysis. GO enrichment analysis for genes on biological processes (BP) revealed that cold-induced DEGs were abundant in the processes of lipid catabolism, negative regulation of coagulation and protein-lipid complex remodeling (Figure 4A). The cnetplot showed that these apolipoprotein encoding genes (*APOC3*, *APOA4*, *APOE*, *APOA2*, *APOC2*, *APOA5*), involved in natural lipid catabolic progress, negative regulation of coagulation, protein-lipid complex remodeling, were significantly upregulated by cold exposure (Figure 4D), suggesting that cold exposure might influence lipid metabolism in LDM through these processes. GO enrichment analysis for genes on molecular function (MF) revealed enrichment in lipid binding, endopeptidase inhibitor activity and extracellular matrix structural constituent (Figure 4B). These upregulated apolipoprotein encoding genes also participated in lipid binding (Figure 4E). These endopeptidase inhibitor activity related genes (*SERPIND1*, *AMBP*, *AHSG*, *SERPINA3-2*) were markedly upregulated by cold exposure (Figure 4C), suggesting that cold treatment induced alteration on lipid metabolism might be entangled with endopeptidase inhibitor activity, which reportedly could improve hepatic steatosis and inflammation (Jiang et al., 2020). GO enrichment analysis for genes on cellular components (CC) revealed that cold-induced DEGs were enriched in the extracellular matrix and lipoprotein particle (Figure 4C). The cneplot showed that lipoprotein particles were regulated by these upregulated apolipoprotein encoding genes, and extracellular matrix was mainly regulated by the matrix metalloproteinase (MMP) family (*MMP11*, *MMP8*, *MMP9*, *MMP2*), fibulin encoding genes (*FBLN5*), and fibrillin encoding gene (*FBN1*) upon cold exposure (Figure 4F). These results highlight the regulatory roles of lipid catabolic progress, lipoprotein complex remodeling and extracellular matrix, especially apolipoproteins, in lipid metabolism of LDM under cold exposure treatment.

### Cold Exposure Induced Alterations in Metabolism and Inflammation Regulatory Pathways

**FIGURE 5:**
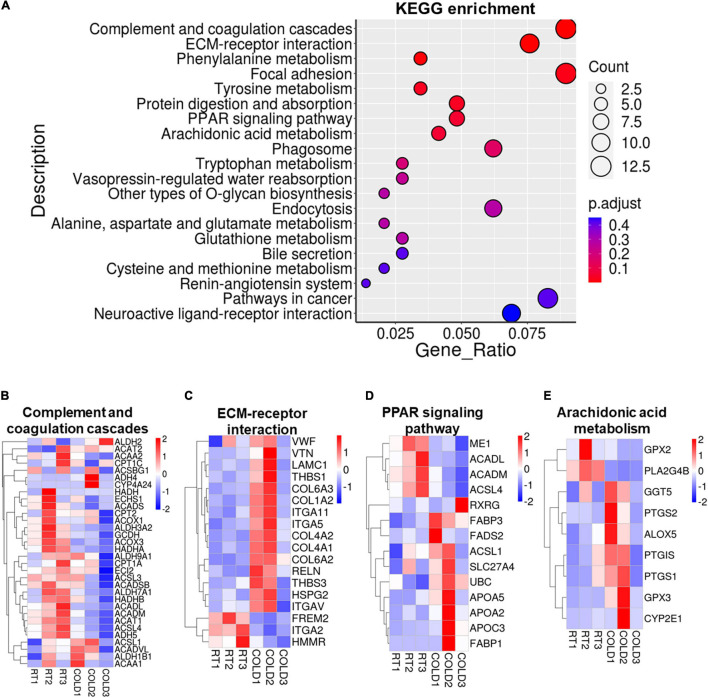
Cold exposure induced alterations in metabolism and inflammation regulatory pathways. **(A)** Functional enrichment analyses for DEGs. **(B–E)** Heatmaps of TPM expression values of Complement and coagulation cascades **(B)**, ECM-receptor interaction **(C)**, PPAR signaling pathways **(D)** and Arachidonic acid metabolism **(E)** regulated genes from the RNA-seq dataset. Only genes with *P* < 0.05 are displayed.

KEGG pathways analysis were applied on these DEGs and revealed most enrichment in complement and coagulation cascades, ECM-receptor interaction, phenylalanine metabolism, focal adhesion, tyrosine metabolism, protein digestion and absorption, and PPAR signaling pathways (Figure 5A). Heatmaps showed the expressions of genes involved in complement and coagulation cascades, ECM-receptor interaction, PPAR signaling pathways and arachidonic acid metabolism (Figures 5B–E). Most genes involved in complement and coagulation cascades were induced by cold exposure in LDM of pigs, especially *ACADL*, *ACADM*, *ACSL3*, *ACSL4* (Figure 5B). These collagen-alpha-proteins encoding genes (*COL4A2*, *COL6A2*, *COL6A3*, *COL4A1*, and *COL1A2*) involved in the ECM-receptor interaction were significantly increased in cold-treated LDM (Figure 5C). The expression level of hyaluronan mediated motility receptor (*HMMR*) whose function blocking reportedly promotes adipogenesis was significantly inhibited by cold exposure in LDM (Figure 5C), which might be partly responsible for the increased trend of IMF content. Cold exposure treatment upregulated the expression levels of fatty acid binding protein encoding genes (*FABP1*, *FABP3*) and apolipoprotein encoding genes (*APOA2*, *APOA5*, *APOC3*), and downregulated mitochondrial fatty acid beta-oxidation related genes (*ACADM*, *ACADL*), which are involved in PPAR signaling pathway (Figure 5D). The expression level of retinoid X receptor gamma (*RXRG*) was increased in LDM of pigs upon cold exposure (Figure 5D). Notably, cold exposure significantly altered the expression of genes involved in arachidonic acid metabolism (Figure 5E). We found that cold exposure elevated the expression levels of *PTGIS*, *PTGS1*, *PTGS2*, *ALOX5*, *GGT5* (Figure 5E). These pathways enrichment results suggested that alterations in meat quality and fatty acid composition of LDM induced by cold exposure might be regulated by both metabolic and inflammatory pathways. Besides, these cold-induced DEGs were also enriched in various amino acids metabolism related pathways, including phenylalanine metabolism, tyrosine metabolism, tryptophan metabolism, alanine, aspartate and glutamate metabolism, glutathione metabolism, cysteine and methionine metabolism, which are also associated with meat nutritional values (Figure 5A). We further examined the amino acid contents in LDM from COLD and RT pigs, but observed no significant difference between these two groups (Supplementary Table 1).

## Discussion

Many studies have reported that cold exposure could induce various alterations on whole-body metabolism in mammals including mice, humans and birds (Chung et al., 2017; Wakabayashi et al., 2017; Nyuiadzi et al., 2020). In our experiment, we investigated the changes in meat quality and nutritional values and further discovered the regulatory mechanisms in the LDM of pigs in response to cold exposure through RNA-seq technology. Our results showed that cold exposure induced significant changes in meat colour_24 h_, pH_24 h_ and fatty acid composition, as well as lead to an increase in IMF. RNA-seq results indicated that cold exposure activated fatty acid anabolism while inhibiting fatty acid catabolism in LDM, which might be regulated by metabolic and inflammatory pathways. Our results suggest that pre-slaughter cold exposure treatment in pigs might improve the meat quality and nutritional values of skeletal muscle.

The “juiciness” and “tenderness” in pork are strongly influenced by the fat deposition and collagens proportions contained within the meat (Weston et al., 2002; Font-I-Furnols et al., 2012). In this study, we observed that the IMF content, fatty acid contents and expression levels of fatty acid metabolism related genes were altered in pigs LDM under pre-slaughter cold exposure as well as collagen synthesis enzymes encoding genes. These findings suggest that cold exposure might improve the pork eating quality through regulating the lipid metabolism and collagens synthesis toward a more beneficial direction. Besides, the water-holding capacity (WHC), the ability of meat to hold all or part of its water, is considered one of the most important trait of product yield and pork quality, and is largely influenced by meat pH. A low meat pH (below 5.8) 45 min after slaughter is often associated with low WHC and pale meat color, resulting in PSE pork. In contrast, high meat pH (above 6.0) 24 h after slaughter often causes DFD pork. The variation in meat pH, WHC and color results from the different post-mortem processes such as muscle metabolism (glycolysis) and conversion rates of glycogen into lactic acid, which are influenced by environmental factors, including breeding conditions, nutrition, transport conditions, stress, weather conditions, and the methods of slaughter (Przybylski et al., 2016). In this study, despite the stress related to a sudden lowering of temperature overnight before slaughter resulted in lower meat colour_24 h_ and pH_24 h_ while drip loss after 24 h was not affected. Also, we observed neither pH_24 h_ values above 6.0 nor pH_45 min_ below 5.8 in any of the COLD pigs or the RT controls. Thus, cold exposure treatment might prove a useful future strategy to improve meat quality.

Pork represents a rich source of lipids, which have been an important topic of discussion for consumers of meat due to the disadvantageous correlation between dietary fat intake and the incidence of various lifestyle disorders, including obesity and cardiovascular diseases (Dugan et al., 2015). Any imbalance in the ratio between PUFAs to SFAs ratio as well as omega-6 (n-6) to omega-3 (n-3) fatty acids ratio have been related to a variety of pathologies, such as cardiovascular, inflammatory diseases, diabetes and autoimmune disorders. This led to several studies suggesting to rebalance the fatty acid profiles of pork by increasing the PUFAs to SFAs ratio and n-3 fatty acids contents while decreasing its n-6: n-3 ratio (Kouba et al., 2003; Corino et al., 2014). In our study, we found that overnight cold exposure significantly increased the contents of SFAs, MUFAs, PUFAs, and n-3 fatty acids, while, the PUFAs to SFAs ratio decreased with the n-6: n-3 ratio being significantly increased. As the majority of these detected fatty acids were increased in cold-treated LDM, we conclude that the alterations in fatty acid composition in LDM were mainly the result of the increased the proportion of IMF due to cold. Previous reviews have pointed out that the fatty acid composition of pork can be regulated by reducing total fat content, due to the fairly constant proportion of PUFA-rich phospholipids in the cell membrane, with at the same time the relatively flexible proportion of SFA-rich triacylglycerol in lipid droplet (Wood et al., 2008; Huang et al., 2014). However, too low IMF content may lead to palatability issues. Thus, some strategies using dietary adjustments to ameliorate pork fatty acid profiles without changing the total fat content were used. We thus speculate that overlapping the dietary influence with pre-slaughter cold exposure treatment in pigs might become an efficient and satisfying method to improve pork quality and its nutritive value. It is useful to compare fatty acid composition in LDM from meat described in the literature. When assessing the nutritional values of pork, it is important to consider site-specific differences, because the fibrous tissue envelope, the epimysium and adipose tissue are extensive constituents of pork pieces cut from different parts, while adipose tissue always contains much more lipids than muscle fibers (Turner et al., 2014; Dugan et al., 2015). Thus, future work will have to address even precisely the effects of cold exposure on fatty acid composition of the adipose tissues on top of skeletal muscle in pigs, especially from the muscle-associated adipose tissue.

Our RNA-seq results revealed that cold exposure significantly upregulated fatty acid biosynthesis related genes (*ACSL1*, *FADS2*, *ELOVL1*, *SCD5*) and downregulated the expression of mitochondrial beta-oxidation related genes (*ACADL*, *ACADM*, *ACAT1*, *ACSL4*) in skeletal muscle, which might directly explain the increased trend of IMF content and alterations of fatty acids in LDM of COLD pigs. In white and brown adipose tissue, cold exposure induces dynamic, heterogeneous alterations in lipid content and lipid metabolism regulatory pathways, especially the dramatically activated fatty acid beta-oxidation (Coolbaugh et al., 2019; Xu Z. et al., 2019). Both skeletal muscle and adipose tissues are considered important metabolic organs, while these two organ systems might play distinct roles in maintaining whole-body homeostasis under cold exposure treatment. The GO enrichment analysis of our RNA-seq results highlighted the alterations of apolipoprotein encoding genes (*APOA2*, *APOA5*, *APOC3*). APOC3 was reported to be among the key proteins regulating the different lipid deposition ability in skeletal muscle from Chinese native mini-type breeds’ pigs and introduced western breeds (Wang et al., 2017). Thus, we concluded that lipoprotein particles might also participate in the regulation of lipid metabolism in skeletal muscle following cold exposure.

KEGG enrichment analysis of our RNA-seq results revealed that DEGs were abundant in complement and coagulation cascades, ECM-receptor interaction, phenylalanine metabolism, focal adhesion, tyrosine metabolism, protein digestion and absorption and PPAR signaling pathway. These upregulated fatty acid binding and transport related genes (*FABP1*, *FABP3*, *SLC27A4*, and *FATP4*) might function in conjunction with fatty acid biosynthesis and oxidation related genes to adapt the lipid metabolism in skeletal muscle upon cold exposure. On the other hand, complement and coagulation are evolutionarily related proteolytic cascades in the blood, which is essential for inflammatory responses (Oikonomopoulou et al., 2012; Conway, 2018). The network of ECM-receptor interaction, focal adhesion, protein digestion and absorption play several roles including but not limited to force transmission, growth factors regulation, inflammatory responses, and muscle stem cell proliferation and differentiation (Mohassel et al., 2018; Sorensen et al., 2018; Lionello et al., 2019). Besides, previous studies demonstrated that reduced temperatures impair glutamine-induced anabolic response in human primary myotubes (Rantala and Chaillou, 2019) while cold acclimation affects L-arginine-modulated antioxidative defense in skeletal muscle (Petrovic et al., 2008). We also found an enrichment of amino acid metabolism pathways, including phenylalanine metabolism, tyrosine metabolism, tryptophan metabolism, alanine, aspartate and glutamate metabolism, glutathione metabolism, cysteine and methionine metabolism in this study. However, the content of amino acids showed no significant difference. The overnight cold exposure treatment may have been too short to cause obvious changes in the content of amino acids or amino acids are somewhat less responsive to compositional changes due to a temperature drop. Taken together, our RNA-seq results revealed various alterations in cold-treated skeletal muscle, with the specific impact and regulatory mechanisms cold exposure on skeletal muscle still unclear.

## Conclusion

In conclusion, our study reveals significant alterations in fatty acid profile and lipid metabolism of pig skeletal muscle upon overnight cold exposure. We carefully evaluated the potential regulations for meat quality and nutritional values of pigs with our experimental test. More detailed investigations uncovering the cold exposure induced specific effects and regulatory mechanisms in skeletal muscles of economically relevant meat should be performed.

## Data Availability Statement

The datasets presented in this study can be found in NCBI under the sample ID are SRR15694341, SRR15694342, SRR15694343, SRR15694344, SRR15694345, and SRR15694346.

## Ethics Statement

The animal study was reviewed and approved by the University of Zhejiang Institutional Animal Care and Use Committee.

## Author Contributions

ZX: investigation, methodology, formal analysis, and writing–original draft. WC and LW: investigation and writing–original draft. YZ and QN: investigation. TV: writing–review and editing. YW and JX: resources. TS: project administration, writing–review and editing, and supervision. All authors have read and approved the final manuscript.

## Conflict of Interest

JX was employed by company Shandong Chunteng Food Co., Ltd. The remaining authors declare that the research was conducted in the absence of any commercial or financial relationships that could be construed as a potential conflict of interest.

## Publisher’s Note

All claims expressed in this article are solely those of the authors and do not necessarily represent those of their affiliated organizations, or those of the publisher, the editors and the reviewers. Any product that may be evaluated in this article, or claim that may be made by its manufacturer, is not guaranteed or endorsed by the publisher.
