# Supplementary material for: Cold Exposure Affects Lipid Metabolism, Fatty Acids Composition and Transcription in Pig Skeletal Muscle
Source: Front Physiol. 2021 Oct 6;12:748801. doi: 10.3389/fphys.2021.748801 (PMC8526723; doi:10.3389/fphys.2021.748801)
Supplement: Supplementary file 1 [file Data_Sheet_1.docx]

**Supplementary Table 1.** Effect of overnight cold exposure on amino acid contents in LDM of pigs.

|  | RT | | COLD | |  |
| --- | --- | --- | --- | --- | --- |
| Variable | Mean | SEM | Mean | SEM | P-value |
| Aspartic acid | 6.77 | 0.23 | 6.45 | 0.35 | 0.46 |
| Threonine | 3.34 | 0.08 | 3.19 | 0.17 | 0.45 |
| Serine | 2.70 | 0.04 | 2.65 | 0.14 | 0.72 |
| Glutamic acid | 10.15 | 0.18 | 9.80 | 0.59 | 0.58 |
| Glycine | 3.11 | 0.13 | 3.02 | 0.18 | 0.68 |
| Alanine | 4.38 | 0.23 | 4.20 | 0.26 | 0.62 |
| Cystine | 0.49 | 0.17 | 0.32 | 0.15 | 0.49 |
| Valine | 3.36 | 0.14 | 3.20 | 0.22 | 0.55 |
| Methionine | 2.20 | 0.08 | 2.06 | 0.11 | 0.32 |
| Isoleucine | 3.38 | 0.17 | 3.19 | 0.23 | 0.50 |
| Leucine | 6.53 | 0.28 | 6.29 | 0.45 | 0.66 |
| Tyrosine | 2.64 | 0.1199 | 2.43 | 0.13 | 0.26 |

Note: Statistical effect of cold exposure on amino acid contents in LDM of pigs were analyzed by two-tailed Student’s t-test (n=6). SEM, standard of error means.

**Supplementary Figure 1.** **Relative mRNA expression levels of the genes in the LDM by cold exposure in pigs.** (A) qPCR validation of the expression of genes related to myofiber type in LDM from cold-treated and RT pigs.
